# MM/PB(GB)SA benchmarks on soluble proteins and membrane proteins

**DOI:** 10.3389/fphar.2022.1018351

**Published:** 2022-12-01

**Authors:** Shiyu Wang, Xiaolin Sun, Wenqiang Cui, Shuguang Yuan

**Affiliations:** ^1^ Research Center for Computer-Aided Drug Discovery, Shenzhen Institute of Advanced Technology, Chinese Academy of Sciences, Shenzhen, China; ^2^ College of Chemical Science, University of Chinese Academy of Sciences, Beijing, China; ^3^ AlphaMol-SIAT Joint Laboratory, Shenzhen, China; ^4^ Faculty of Chemistry, University of Warsaw, Warsaw, Poland

**Keywords:** MM/PB(GB)SA, FEP, binding energy, membrane protein, soluble protein

## Abstract

Predicting protein-ligand binding free energy rapidly and accurately remains a challenging question in modern drug discovery. Molecular mechanics/Poisson-Boltzmann (Generalized Born) surface area (MM/PB(GB)SA) has emerged as an essential tool for accelerating cost-efficient binding free energy calculation. This study presents benchmarks with three membrane-bound protein systems and six soluble protein systems. Different parameters were sampled for different benchmarks to explore the highest accuracy. These include ligand charges, protein force fields, extra points, GB models, nonpolar optimization methods, internal dielectric constants and membrane dielectric constants. Comparisons of accuracy were made between MM/PB(GB)SA, docking and free energy perturbation (FEP). The results reveal a competitive performance between MM/PB(GB)SA and FEP. In summary, MM/PB(GB)SA is a powerful approach to predict ligand binding free energy rapidly and accurately. Parameters of MM/PB(GB)SA calculations, such as the GB models and membrane dielectric constants, need to be optimized for different systems. This method can be served as a powerful tool for drug design.

## Introduction

Free energy plays essential roles in biological events, such as protein folding, enzyme catalysis and target-drug binding (Wang et al., 2019). Therefore, predicting binding free energy accurately is of great importance in related research, especially in drug discovery (Jorgensen, 2004). Free energy perturbation (FEP) (Jorgensen, 1989), thermodynamic integration (TI) (Kirkwood, 1935; Genheden et al., 2011) and molecular mechanics Poisson-Boltzmann (Generalized Born) surface area (MM/PB(GB)SA) (Still et al., 1990; Srinivasan et al., 1998; Kuhn and Kollman, 2000) are the most commonly used computational methods for calculating the binding free energy. FEP and TI, which are pathway-based methods, have been considered more accurate tools than MM/PB(GB)SA. However, the application in research is often hampered by slow convergence, complicated system building and huge computational costs (Wang et al., 2019). To address the limitation, MM/PB(GB)SA, an end-point method, has become an attractive approach for calculating binding free energy (ΔG_bind_) between protein and ligand.

As implicit continuous solvation models, the GB and PB models are designed to predict the solvation free energy change in the binding process. In MM/PB(GB)SA method, the binding free energy in solvation (ΔG_bind, solve_) could be decomposed into the binding free energy in vacuum (ΔG_bind, vacuum_) and solvation free energy (ΔG_solve_) (Srinivasan et al., 1998):

$${\Delta G}_{bind,\;solve}={\Delta G}_{bind,\;vacuum}+{\Delta G}_{solve}$$

$$={\Delta E}_{MM}+{\Delta G}_{solve}-T\Delta S$$

$$={\Delta E}_{MM}+{\Delta G}_{PB\left(GB\right)}+{\Delta G}_{SA}-T\Delta S$$ (1)

where ΔE_MM_ is the molecular interaction energy, which includes bond energy contribution (bond, angle and dihedral energies) and nonbonded energy contribution (electrostatic energies and van der Waals energies). ΔG_PB(GB)_ and ΔG_SA_ are the polar and nonpolar energy contributions of ΔG_solve_, in which, ΔG_SA_ is directly proportional to solvent-accessible surface area (SASA). -TΔS is conformational entropy contribution.

Compared to FEP and TI, MM/PB(GB)SA has many advantages including high speed, computationally low-cost, user-friendly and stable. By extracting snapshots from MD simulations, ΔG _MM/PB(GB)SA_ can be automatically computed by existing tools such as MMPBSA. py (Miller et al., 2012), g_mmpbsa (Kumari et al., 2014) and gmx_MMPBSA (Valdés-Tresanco et al., 2021). Meanwhile, the disadvantages are also obvious: Because of the large error and high computational cost, the entropy term is neglected in practice, which may reduce the accuracy of the MM/PB(GB)SA approach if the system is an entropy-driven process. As an end-point approach, MM/PB(GB)SA ignores the kinetic pathway, which also contributes to drug activity during drug-target interaction (Wang et al., 2019). In addition, the conformations of protein and ligand extracted from the complex are approximately regarded as their free conformations in the single-trajectory approach (Sham et al., 2000), but the conformations may change during the binding process. Despite these limitations, MM/PB(GB)SA is still one of the most popular approaches for predicting binding free energy. More recently, researchers modified the standard MM/PB(GB)SA method for improving the performance in predicting the binding energy in both protein-protein complexes and protein-ligand complexes, including the screening electrostatic energy (Sheng et al., 2021; Zhu et al., 2022) and interaction entropy (Duan et al., 2016), further making MM/PB(GB)SA more accurate and popular in binding free energy calculation task.

Previous works (Hou et al., 2011b; Su et al., 2015; Sun et al., 2018) have thoroughly investigated the effects of force field, simulation length, sampling method and entropy on the performance of MM/PB(GB)SA. The results imply that the performance of MM/PB(GB)SA is case-by-case. There are no universal parameters that can ensure the accuracy of the prediction in all systems. Moreover, the halogen bond, an important molecular interaction should be considered in MM/PB(GB)SA calculations (Nunes et al., 2019; Fortuna and Costa, 2021). Most of the works mainly focus on soluble protein systems. As the most important category of drug targets, membrane protein systems have not been discussed intensively yet.

Here, we have systemically investigated the effect of model parameters on the performance of MM/PBSA and MM/GBSA methods in both soluble as well as membrane protein systems. In this work, we compared the results between MM/PB(GB)SA, FEP and docking. As a result, MM/PB(GB)SA showed comparable accuracy with FEP, whereas docking showed the worst outcome. MM/PB(GB)SA is a powerful approach for accelerating the accurate prediction of protein-ligand binding free energy. Parameters of MM/PB(GB)SA calculations need to be benchmarked for a specific system. The high accuracy of MM/PB(GB)SA suggests that this method can be applied to virtual screening and lead optimization accurately and efficiently.

## Materials and methods

### Preparation of complexes

The benchmarks were performed on testing systems with 140 ligands bound to six soluble proteins as well as 37 ligands bound to three membrane proteins. The soluble proteins were selected from a public benchmark dataset organized by Schrodinger Inc. for evaluating FEP prediction (Wang et al., 2015). Although eight systems were provided in this dataset, we only selected cyclin-dependent kinase 2 (CDK2), Tyrosine kinase 2 (TYK2), p38 mitogen activated protein kinases (P38), Myeloid Cell Leukemia 1 (Mcl1), c-Jun N-Terminal Kinase 1 (Jnk1) and thrombin systems. This is mainly because the FEP method showed the best performance in the TYK2 system, the worst performance in the CDK2 system and average performances in P38, Mcl1, Jnk1 and thrombin systems. Three membrane complex systems were also tested in this study, including microsomal prostaglandin E synthases (mPGES), G-protein-coupled bile acid receptor (GPBAR) and orexin 1 (OX1). GPBAR and OX1 belong to G protein-coupled receptor (GPCR) superfamily which is the most important drug target. Different from the soluble proteins’ ligands, the ligands of GPCR are divided into agonists and antagonists. We selected 13 agonists as the ligands of GPBAR and 12 antagonists as the ligands of OX1.

To make sure the ligands are bound to protein with the suitable conformations, ligands were docked into the pocket with reference ligands as constraints by Glide SP software (Friesner et al., 2004). The constraint method restricts the maximum common substructure position between ligands and reference molecules. The reference ligands were retrieved from crystal structures with IDs 1H1Q for CDK2, 3FLY for P38, 2ZFF for Thrombin, 4GIH for Tyk2, 2GMX for Jnk1, 6HW3 for Mcl1, 5TL9 for mPGES, 7CFM for GPBAR, 4ZJ8 for OX1. After docking, the conformations of ligands were manually confirmed and selected. The protonated states of ligands and proteins were generated by Schrodinger 2021v1 software at a pH of 7.0. The activity values of ligands were obtained from prior publications (Hardcastle et al., 2004; Szczepankiewicz et al., 2006; Baum et al., 2009; Goldstein et al., 2011; Friberg et al., 2013; Liang et al., 2013; Piotrowski et al., 2013; Roecker et al., 2014; Partridge et al., 2017). The experimental binding free energies of the ligands were calculated by the following approximation (Wang et al., 2015):

$$\Delta G_{\exp}=RTlnK$$ (2)

where T = 297 K, R is the gas constant and K represents the value of affinity, which can be IC_50_, Ki or Kd in nM in our case. Although IC_50_ cannot be a representation of binding affinity directly, it can be converted to Ki with the Michaelis-Menten equation, indicating that IC_50_ and Ki are linearly correlated with constant concentrations of protein and ligand. Therefore, IC_50_ can also be used in Eq. 2.

**FIGURE 1:**
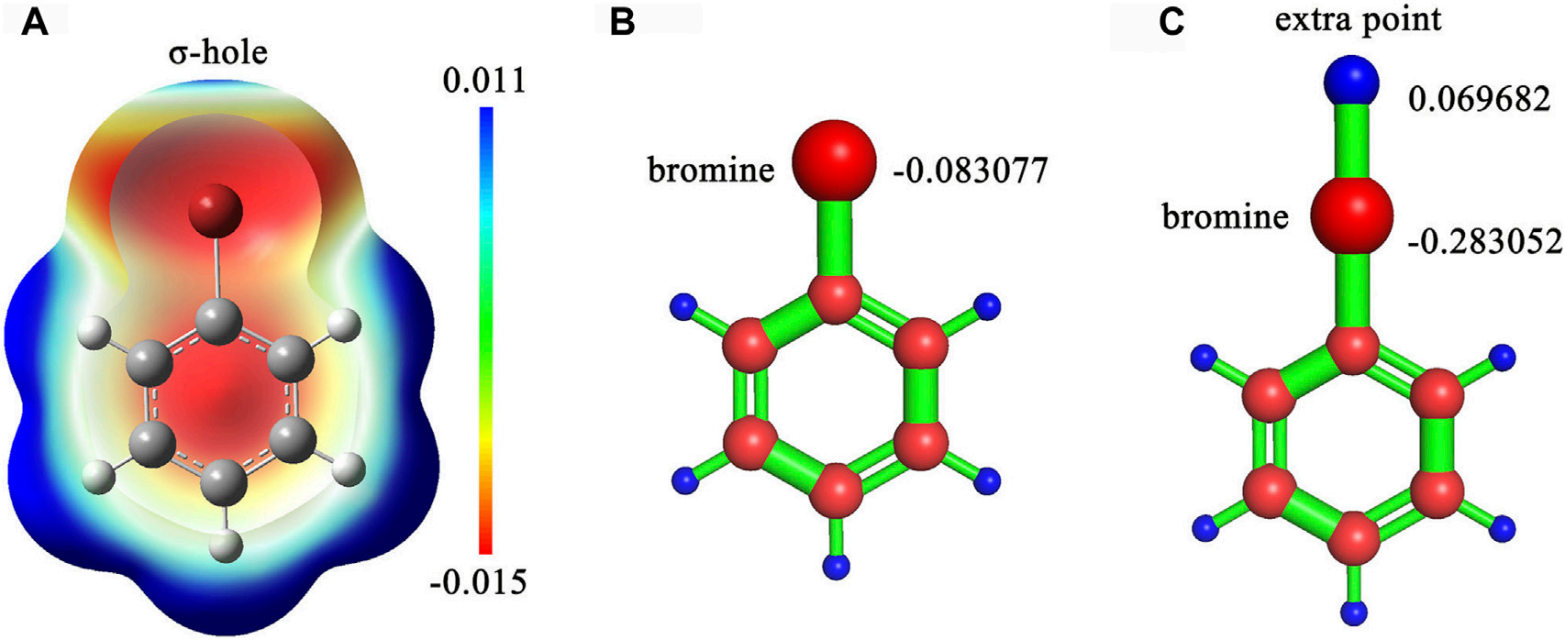
**(A)** The sigma-hole effect in bromobenzene molecule. **(B)** The RESP charge on the bromine atom without an extra point in bromobenzene. **(C)** The RESP charges on the bromine atom and the extra point in bromobenzene. The calculation was processed at B3LYP/6-311G (d,p) level.

**FIGURE 2:**
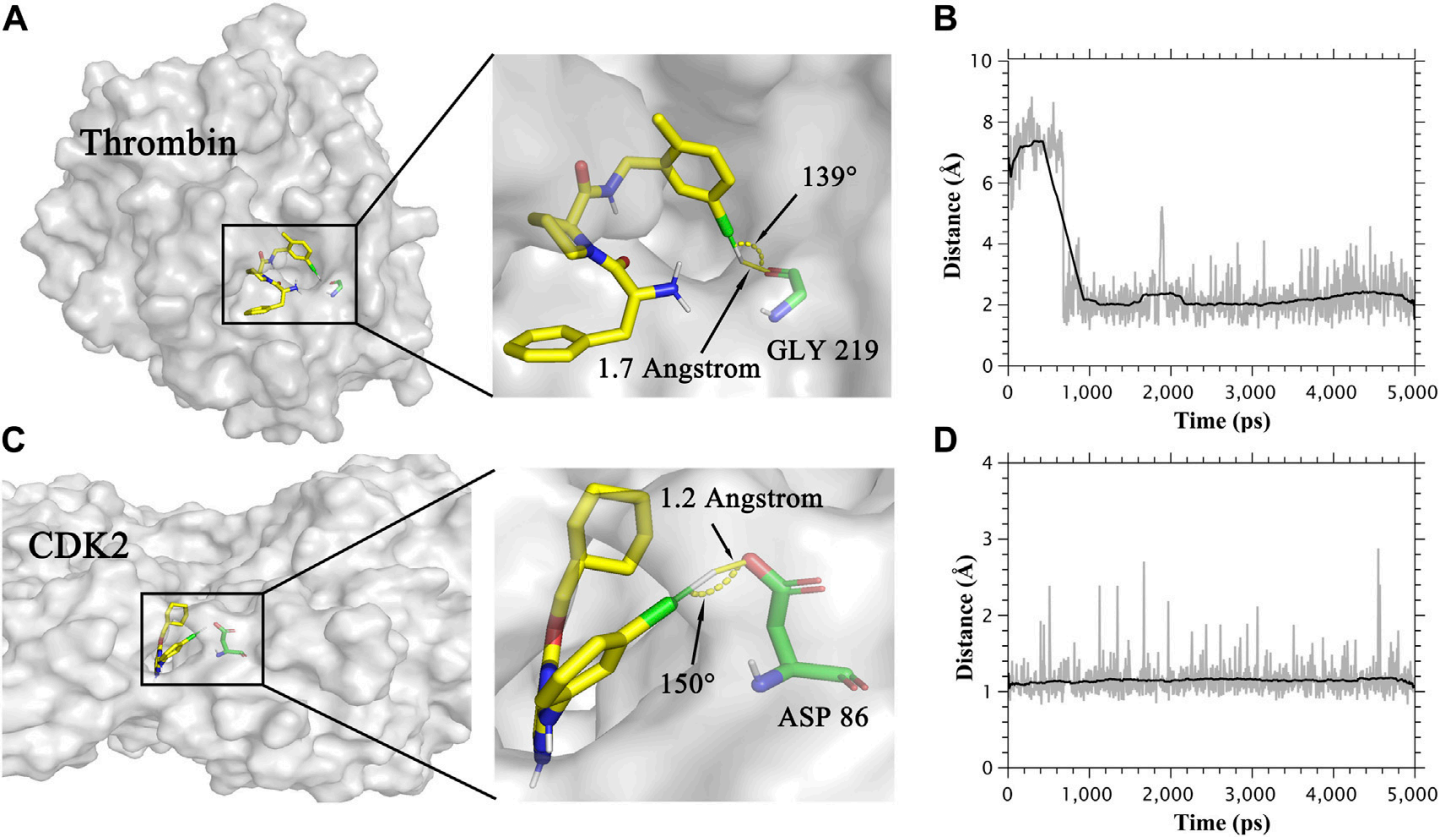
**(A)** The binding mode of Thrombin protein and its ligand. **(B)** Distance between the oxygen atom and extra point in the halogen bond in the Thrombin system. **(C)** The binding mode of CDK2 protein and its ligand. **(D)** Distance between the oxygen atom and extra point in the halogen bond in the CDK2 system.

Six ligand charge methods were adopted in this study, consisting of CHARMM General Force Field (CGenFF) charge (Vanommeslaeghe et al., 2010), AM1-BCC charge, restrained electrostatic potential (Bayly et al., 1993) with Hartree-Fock theory (RESP-HF) charge, RESP with Density-functional theory (RESP-DFT) charge, RESP-HF with extra points (RESP-HF-EP) charge and RESP-DFT with extra points (RESP-DFT-EP) charge. CGenFF charge was generated by CGenFF program version 2.5 (Vanommeslaeghe et al., 2010). Where, the extra point is a dummy atom with positive charges on the extension line of carbon halogen bond for simulating the halogen bond interaction in molecular dynamics (Figure 1). AM1-BCC charge was generated by the antechamber and sqm program in AmberTools 2020. For RESP-HF charge, geometry optimization and single-point electrostatic potential calculation were performed at HF/6-31G(d) level, which is compatible with the Amber force field. For RESP-DFT charge, geometry optimization and single-point electrostatic potential calculation were performed at B3LYP/6-311G (d,p) level. Unlike the classical RESP charges, RESP-HF-EP and RESP-DFT-EP charges couldn’t be fitted by Antechamber, because the coordinates of extra points needed to be determined manually. Therefore, CGenFF was applied to determine the coordinates of extra points. The resp program in AmberTools2020 was used to refit the atomic charges after single-point electrostatic potential calculation for RESP-HF-EP and RESP-DFT-EP charges. Other parameters of extra points were also generated by CGenFF, including bond length, bond angle and dihedral angle. For main family elements in or after the fourth cycle of the periodic table of elements, the SDD basis set was applied in quantum chemical calculation. Density functional dispersion correction (Grimme et al., 2010) (DFT-D3) was also applied to simulate dispersion interaction. All quantum chemical calculations about RESP charges were finished by Gaussian 09. The Polarizable Continuum Model (PCM) (Tomasi et al., 2005) implicit water model was used in QM calculation for simulating the real solvation condition of molecules. More detailed information about calculating RESP-HF-EP and RESP-DFT-EP charges can also be found in our repository (https://github.com/shiyu-wangbyte/MM-PB-GB-SA_Benchmarks). An example of the extra point is drawn in Figure 1. The graphs of extra points in CDK2 and Thrombin systems can be found in Figures 2A,C.

The setting up of the soluble protein complexes was performed by GROMACS(Bekker et al., 1992) 2020. Two different protein force fields amber FF99SB and charmm36m (Huang et al., 2017) were applied for proteins. For better performance in MM/PBSA calculations, we used Amber FF99SB force field (Xu et al., 2013). For ligands with AM1-BCC charge, RESP-HF charge and RESP-DFT charge, a general AMBER force field (Wang et al., 2004) (GAFF) was applied. For ligands with CGenFF charge, RESP-HF-EP charge and RESP-DFT-EP charge, CHARMM General Force Field was applied. TIP3P water model was used to wrap complexes extending 1.2 nm away from the edges of the complexes. 0.15 M NaCl was also added to neutralize the whole system The setting up of the membrane-bound protein complexes was performed by CHARMM-GUI (Jo et al., 2008) and GROMACS 2020. The 1,2-palmitoyl-oleoyl-sn-glycero-3-phosphocholine (POPC) model was added for membrane simulation by CHARMM-GUI. The charmm36 m lipid force field (Huang et al., 2017) and the amber lipid14 force field (Dickson et al., 2014) were used to parameterize membrane molecules when the charmm36 m force field and FF99SB were applied to proteins, respectively. Like soluble protein complexes, the TIP3P water model and 0.15 M NaCl were added to membrane-bound protein systems.

### Molecular dynamics simulation

MD simulation consisted of energy minimization, pre-equilibration and production. For soluble protein systems, all molecules were energy-minimized within 5,000 steps of steepest descent while keeping the solute atoms restrained at a force constant of 1000 KJ/(mol*nm^2^). The system was then heated to 297 K during a 300 ps simulation in constant volume and temperature (NVT) condition subsequently followed by a 400 ps simulation in constant pressure and temperature (NPT) condition where solute atoms were subjected to 1000 KJ/(mol*nm^2^). Finally, 5ns production simulation was performed under NPT conditions where backbone atoms were subjected to 300 KJ/(mol*nm^2^). The nonbonded interaction cutoff was set to 1.2 nm during the whole simulation. MD simulation of membrane-bound protein complexes was more complex. Systems were also energy minimized within 5,000 steps of steepest descent while keeping 4000 KJ/(mol*nm^2^) force constant on backbone atoms and ligand atoms, keeping 2000 KJ/(mol*nm^2^) force constant on side-chain atoms and keeping 1000 KJ/(mol*nm^2^) force restraint on lipid atoms. Then, six steps NPT simulations (300, 300, 500, 250, 250, and 250 ps) were carried out, where restraint was reduced slowly (4,000, 2000, 1,000, 500, 300, and 300 KJ/(mol*nm^2^) on the backbone and ligand atoms, 2000, 1,000, 500, 200, 50, and 50 KJ/(mol*nm^2^) on side-chain atoms, 1,000, 400, 400, 200, 40, and 0 KJ/(mol*nm^2^) on lipid atoms, respectively) to relax the system. Finally, a 5 ns NPT production simulation was carried out with a force constant of 300 KJ/(mol*nm^2^) on backbone atoms. The position restraints were applied during the equilibration phase to avoid drastic rearrangements of critical parts. As the binding conformations of protein and ligand are known, position restraints were applied during the production phase for reducing the amplification of force field errors in long-term molecule dynamics, which is the reason why longer molecule dynamics does not contribute to the accuracy of the MM/PBSA calculation (Hou et al., 2011b; Su et al., 2015). All MD simulations were performed by GROMACS 2020 software.

### MM/PB(GB)SA calculations

Gmx_MMPBSA (Valdés-Tresanco et al., 2021) Version1.4.3 and MMPBSA. py (Miller et al., 2012) were used to compute MM/PB(GB)SA. 100 frames were taken evenly from the MD trajectory from 3 to 5 ns for calculating MM/PB(GB)SA. In this study, we discussed the performance of five different GB models in membrane-bound protein systems, including the pairwise model GB^HCT^ (igb = 1) (Hawkins et al., 1996), the modified GB model GB^OBC^ (igb = 2) (Onufriev et al., 2000), the optimized version GB^OBC2^ (igb = 5) (Onufriev et al., 2004), the GBneck model to solve the “bottleneck” issue (igb = 7) (Mongan et al., 2007) and the optimized version GBneck2 (igb = 8) (Nguyen et al., 2013). A fast LCPO algorithm (Weiser et al., 1999) was used to estimate solvent accessible area in MM/GBSA calculation. In the MM/PBSA calculation of solvation protein systems, the ionic strength was set to 0.15 M. Other parameters were default values in MMPBSA. py. For example, the exterior dielectric constant of 80 and solute dielectric constant of 1 were used. As the application of the implicit membrane model, the MM/PBSA calculation in membrane-bound protein systems was different from that in solvation protein systems. As a partially polar solvent, the membrane could also affect the ligand-binding process. The parameters set of MM/PB(GB)SA calculation in membrane-bound protein systems followed the instruction of the Amber reference manual. As an example, the ratio between the longest dimension of the rectangular finite-difference grid and that of the solute of 1.25 and the internal dielectric constant of 20 were set. More parameters set in MM/PB(GB)SA calculation can be found in the Supplementary Material S1. After that, the effect of the internal dielectric constant, membrane dielectric constant and nonpolar optimization method were also discussed. In addition, we deleted the extra points and added the charge on extra points back to halogen atoms in trajectory and topology files, because MMPBSA. py cannot process molecules with extra points.

Pearson correlation coefficient (R) and mean absolute error (MAE) were used to evaluate the performance of MM/PB(GB)SA in our testing systems. R and MAE were used to characterize the degree of linear correlation and the real error between MM/PB(GB)SA and ΔG_exp_, respectively. Because MM/PB(GB)SA cannot be compared with ΔG_exp_ directly, MM/PB(GB)SA needs to be linear fitted and transformed before MAE calculation (see Supplementary Material S1).

## Results

To monitor the system stability during MD simulation, the conformational Root Mean Squared Distance (RMSDs) of receptors and ligands are shown in Supplementary Figure S1. All RSMDs were less than 2 Angstrom during the whole production simulation, implying that the conformations of ligands and receptors do not change sharply. RMSDs of receptors’ backbone atoms were less than that of ligands in all nine systems, which was because 300 KJ/(mol*nm^2^) restrain was applied to the backbone atoms of proteins. In CDK2, TYK2, Mcl1, mPGES, GPBAR and OX1 systems, receptors and ligands were in a state of equilibrium during the whole production simulation. In P38, Jnk1 and thrombin systems, ligands become stable from 2 ns, 3ns and 3ns on, respectively. Therefore, 3–5ns trajectories were extracted for MM/PB(GB)SA calculations.

### Ligand charge method, protein force field and MM/PB(GB)SA

Combinations of four different ligand charge methods and two protein force fields were compared in this section. The Pearson correlation coefficient (R) and mean absolute error (MAE) of experimental and predicted binding free energies of those combinations on single systems were shown in Table 1. The total performance of these combinations on soluble protein systems, membrane protein systems and all protein systems was also shown in Supplementary Table S1. The R of combination of the CGenFF charge method and charmm protein force field were -0.17 for MM/GBSA and -0.09 for MM/PBSA, which were the lowest among all combinations. The R of the AM1-BCC charge and FF99SB combination achieved the highest R (0.40) in MM/GBSA calculation, while the combination of RESP_DFT charge method achieved the highest R (0.17) in MM/PBSA calculation. What’s is more, the FF99SB protein force field performed better than the charmm force field with the RESP charge method, although the difference is slight. For example, the R of RESP_HF charge method with FF99SB in MM/PBSA computation was 0.13, which was higher than that of charm force field (0.09). For each single system in Table 1, MM/GBSA showed the highest R in CDK2 (0.78), p38 (0.70) Jnk1 (0.55) and OX1 (0.85) systems with the charge method of RESP_DFT. MM/PBSA showed the highest R in CDK2 (0.52) and OX1 (0.73) systems with the charge method of RESP_DFT. MM/GBSA only showed the highest R in the GPBAR (0.69) and mPGES (0.85) systems with the charge method of RESP_HF. MM/PBSA showed the highest R in the Tyk2 (0.87), Jnk1 (0.56) and GPBAR(0.69) systems with the charge method of RESP_HF. MM/GBSA only showed the highest R in the Mcl1 (0.78) system with the charge method of AM1_BCC. MM/PBSA only showed the highest R in the mPGES (0.87) system with the charge method of AM1_BCC. MM/GBSA only showed the highest R in the Thrombin (0.96) system with the charge method of CGenFF. MM/PBSA showed the highest R in the p38 (0.18), Mcl1 (0.70) and GPBAR (0.76) system with the charge method of CGenFF. In most cases, MM/PB(GB)SA performed better with the FF99SB force field than the CHARMM force field. For example, the R of the combination of FF99SB and RESP_DFT is 0.78 with MM/GBSA method in CDK2 system, which is higher than the counterpart with MM/PBSA calculation. Especially, the most negative correlations were calculated by MM/PBSA in Table 1.

**TABLE 1:** The Pearson correlation coefficient (R) and mean absolute error (MAE) between MM/PB(GB)SA-predicted binding free energies and experimental data based on different ligand charge methods and protein force fields.

		obs. R (higher is better)						MAE (lower is better)					
		RESP_DFT		RESP_HF		AM1_BCC	CGenFF	RESP_DFT		RESP_HF		AM1_BCC	CGenFF
		FF99SB	CHARMM	FF99SB	CHARMM	FF99SB	CHARMM	FF99SB	CHARMM	FF99SB	CHARMM	FF99SB	CHARMM
CDK2	MM/GBSA	0.78	0.51	0.60	0.41	−0.47	0.30	0.83	1.71	1.40	2.28	1.84	2.88
	MM/PBSA	**0.52**	0.31	0.27	0.25	−0.12	−0.22	1.64	3.07	3.64	3.53	8.63	4.48
P38	MM/GBSA	0.65	**0.70**	0.57	0.61	0.65	0.35	0.93	0.83	1.18	1.00	0.96	2.05
	MM/PBSA	−0.14	−0.01	0.01	−0.02	−0.05	0.18	5.86	82.96	101.3	35.62	15.27	4.63
Thrombin	MM/GBSA	0.84	0.73	**0.69**	0.46	0.63	**0.96**	0.26	0.42	0.42	0.75	0.56	0.12
	MM/PBSA	0.26	0.21	0.71	0.23	0.59	0.81	1.30	1.91	0.35	1.84	0.61	0.31
Tyk2	MM/GBSA	0.57	0.62	0.56	0.59	0.52	0.22	2.31	1.41	1.75	1.46	1.66	4.61
	MM/PBSA	0.68	0.86	0.79	0.87	0.76	0.37	1.13	0.62	0.85	0.54	0.78	2.51
Mcl1	MM/GBSA	0.68	0.72	0.6	0.71	**0.78**	0.71	0.87	0.75	1.1	0.88	0.69	0.83
	MM/PBSA	0.62	0.54	0.42	0.48	0.68	**0.70**	1.00	1.33	1.78	1.51	0.88	0.85
JNK1	MM/GBSA	0.55	0.43	0.35	0.25	0.44	0.24	1.04	1.55	1.82	2.64	1.45	2.79
	MM/PBSA	0.18	0.33	0.05	**0.56**	0.08	0.13	3.77	1.95	12.19	1.03	9.17	4.66
mPGES	MM/GBSA	0.83	0.84	0.85	0.82	0.85	0.08	0.33	0.29	0.30	0.34	0.29	6.85
	MM/PBSA	0.80	0.80	0.77	0.67	0.87	−0.15	0.35	0.41	0.35	0.59	0.27	3.58
GPBAR	MM/GBSA	0.66	0.68	0.67	0.69	0.67	0.62	0.95	0.85	0.90	0.89	0.90	0.96
	MM/PBSA	0.69	0.71	0.67	**0.69**	0.63	0.76	0.86	0.89	0.94	0.91	0.95	0.62
OX1	MM/GBSA	**0.85**	0.64	0.58	0.70	0.69	0.75	0.49	1.18	1.30	0.92	0.97	0.85
	MM/PBSA	**0.73**	0.71	0.70	0.66	0.57	0.66	0.75	0.78	0.95	1.14	1.36	1.19

The bold value is the values mentioned in the main text.

### The effect of extra points

In Table 1, the combination of the CGenFF charge and the CHARMM force field showed the highest R in both MM/PBSA and MM/GBSA models for the thrombin system, which was not in accordance with other systems. To figure out the reason why this combination performs best in the thrombin system, we analyzed the trajectories of the thrombin protein and its ligands. As a result, ligands formed halogen bonds with the main chain oxygen atom of GLY219 in thrombin protein. The halogen bond between thrombin protein and one of its ligands was shown in Figure 2A. The distance of this halogen bond was also counted and shown in Figure 2B. Similarly, the halogen bonds between ligands and side-chain oxygen atoms of ASP 86 in the CDK2 system were observed. The halogen bond in the CDK2 system and its distance were also indicated in Figures 2C,D. Therefore, describing halogen bonds properly during molecular dynamics simulation contributed to the accuracy of MM/PB(GB)SA calculation.

To further investigate the role of halogen bonds in MM/PB(GB)SA calculation, extra points of halogen atoms were manually added in RESP-HF-EP and RESP-DFT-EP charge methods (see method section). The MM/PB(GB)SA performance with RESP-HF, RESP-DFT, RESP-HF-EP and RESP-DFT-EP charge methods was shown in Table 2. It can be seen that the R of MM/PB(GB)SA with extra points was higher in CDK2 and thrombin systems. As an example, the R of MM/GBSA with CHARMM protein force field and RESP_HF ligand charge method was increased from 0.41 to 0.77 in the CDK2 system. Meanwhile, in other systems with no halogen bond, no obvious difference in MM/PB(GB)SA performance was found with/without extra points.

**TABLE 2:** The Pearson correlation coefficient (R) and mean absolute error (MAE) with/without adding extra points.

		obs. R (higher is better)								MAE (lower is better)							
		RESP_DFT		RESP_HF		RESP_DFT_EP		RESP_HF_EP		RESP_DFT		RESP_HF		RESP_DFT_EP		RESP_HF_EP	
		FF99SB	CHARMM	FF99SB	CHARMM	FF99SB	CHARMM	FF99SB	CHARMM	FF99SB	CHARMM	FF99SB	CHARMM	FF99SB	CHARMM	FF99SB	CHARMM
CDK2	MM/GBSA	0.78	0.51	0.60	**0.41**	0.82	0.80	0.65	**0.77**	0.83	1.71	1.40	2.28	0.73	0.73	1.23	0.78
	MM/PBSA	0.52	0.31	0.27	0.25	0.49	0.40	0.47	0.58	1.64	3.07	3.64	3.53	1.78	2.31	1.91	1.42
P38	MM/GBSA	0.65	0.70	0.57	0.61	0.65	0.68	0.57	0.60	0.93	0.83	1.18	1.00	0.95	0.85	1.17	1.01
	MM/PBSA	−0.14	−0.01	0.01	−0.02	−0.13	0.02	0.03	0.01	5.86	82.96	101.30	35.62	6.13	37.68	29.06	76.19
Thrombin	MM/GBSA	0.84	0.73	0.69	0.46	0.87	0.76	0.65	0.45	0.26	0.42	0.42	0.75	0.24	0.35	0.51	0.90
	MM/PBSA	0.26	0.21	0.71	**0.23**	0.71	−0.10	−0.02	**0.51**	1.30	1.91	0.35	1.84	0.45	4.20	15.47	0.70
Tyk2	MM/GBSA	0.57	0.62	0.56	0.59	0.66	0.42	0.66	0.30	2.31	1.41	1.75	1.46	1.15	2.19	1.15	3.29
	MM/PBSA	0.68	0.86	0.79	0.87	0.84	0.72	0.84	0.67	1.13	0.62	0.85	0.54	0.70	1.02	0.70	1.11
Mcl1	MM/GBSA	0.68	0.72	0.6	0.71	0.6	0.71	0.55	0.67	0.87	0.75	1.1	0.88	1.06	0.83	1.2	0.98
	MM/PBSA	0.62	0.54	0.42	0.48	0.39	0.06	0.27	0.09	1.00	1.33	1.78	1.51	1.82	14.08	2.94	9.02
Jnk1	MM/GBSA	0.55	0.43	0.35	0.25	0.59	0.40	0.28	0.22	1.04	1.55	1.82	2.64	0.92	1.52	2.33	2.89
	MM/PBSA	0.18	0.33	0.05	0.56	0.23	0.34	0.03	0.42	3.77	1.95	12.19	1.03	2.84	1.83	23.95	1.53
mPGES	MM/GBSA	0.83	0.84	0.85	0.82	0.84	0.70	0.86	0.70	0.33	0.29	0.30	0.34	0.31	0.41	0.30	0.49
	MM/PBSA	0.80	0.80	0.77	0.67	0.83	0.59	0.82	0.52	0.35	0.41	0.35	0.59	0.31	0.75	0.33	0.89

The bold value is the values mentioned in the main text.

### Different GB models in membrane-bound protein systems

In this section, we computed MM/GBSA with five different GB models, including GB^HCT^, GB^OBC^, GB^OBC2^, GBneck and GBneck2 models. The R and MAE of MM/GBSA calculations between the experimental and predicted binding free energies with different GB models in three membrane-bound protein systems were shown in Supplementary Table S2. In most cases, the GBneck model performed best in three test membrane-bound protein systems. GB^HCT^ and GBneck models performed best in the mPGES system and the GBneck model performed best in GPBAR and OX1 systems. As an example, the GBneck model got the highest average R (0.81) and lowest MAE (0.68) in the OX1 system. Meanwhile, GB^HCT^ and GB^OBC^ models also recorded the lowest MAE in the GPBAR system.

### Different nonpolar optimization methods in membrane-bound protein systems

In this section, two methods computing nonpolar solvation energy are compared in MM/PBSA calculations. The results were shown in Supplementary Table S3. The first method (inp = 1) showed higher R and lower MAE in the mPGES system. The average R with this method was 0.81, which was bigger than that with the second method (0.64). The second method (Tan et al., 2007) (inp = 2) showed higher R and lower MAE in OX1 system, while it showed similar R and MAE in GPBAR system.

### Different dielectric constant in membrane-bound protein systems

**FIGURE 3:**
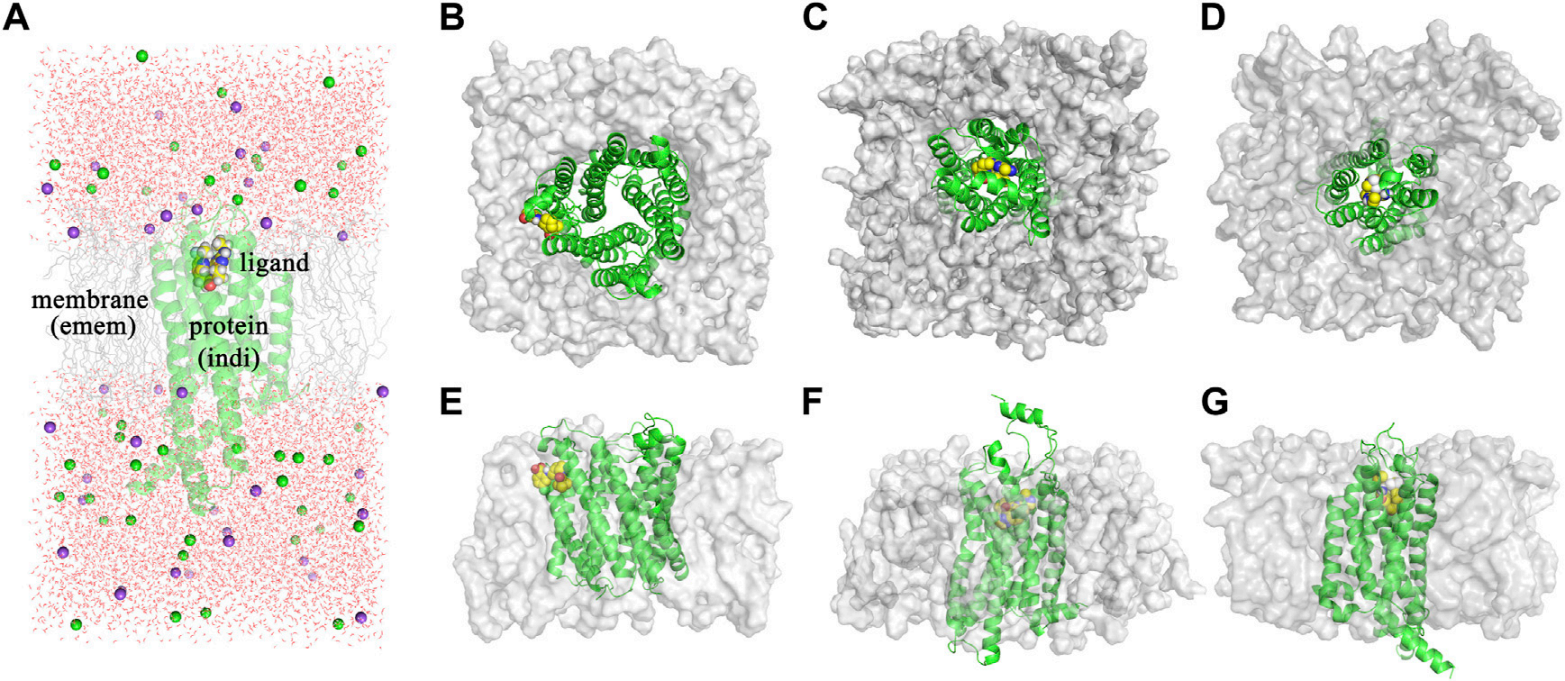
**(A)** The dielectric regions in GPBAR systems. **(B)** The complex structure of mPGES protein and its ligand. **(C)** The complex structure of OX1 protein and its ligand. **(D)** The complex structure of GPBAR protein and its ligand. **(E)** The side view of the mPGES complex structure. **(F)** The side view of the OX1 complex structure. **(G)** The side view of the GPBAR complex structure.

The dielectric regions in Membrane-Bound Protein Systems are shown in Figure 3A. The accuracy of MM/PBSA with different membrane dielectric constants (emem) was shown in Supplementary Table S4. As the membrane dielectric constant was recommended at 7.0 by the Amber reference manual, the gradient of membrane dielectric constants was set as 1.0, 3.0, 5.0, 7.0, and 9.0. In the mPGES system, the MM/PBSA with membrane dielectric constant set to 1.0 showed slightly lower R (0.80) and higher MAE (0.39) than MM/PBSA with other membrane dielectric constants. In GPBAR and OX1 systems, the MM/PBSA with different membrane dielectric constants showed the same correlation coefficient and mean absolute error. The performance of MM/PBSA with different internal dielectric constants (indi) was shown in Supplementary Table S5. The appropriate internal dielectric constant recommended by the Amber reference manual was 20.0. Thus, the gradient of the internal dielectric constant was set as 1.0, 5.0, 10.0, 20.0, and 30.0 in this study. As expected, the performance of MM/PBSA became better with the increase of the internal dielectric constant in three membrane-bound protein systems. For example, the average R with indi = 1 (0.60) was noticeably lower than that with indi = 20 (0.81) in the mPGES system. The growth trend became less obvious when the internal dielectric constant was greater than 5.

### Comparisons among docking, MM/GBSA, MM/PBSA and free energy perturbation

**FIGURE 4:**
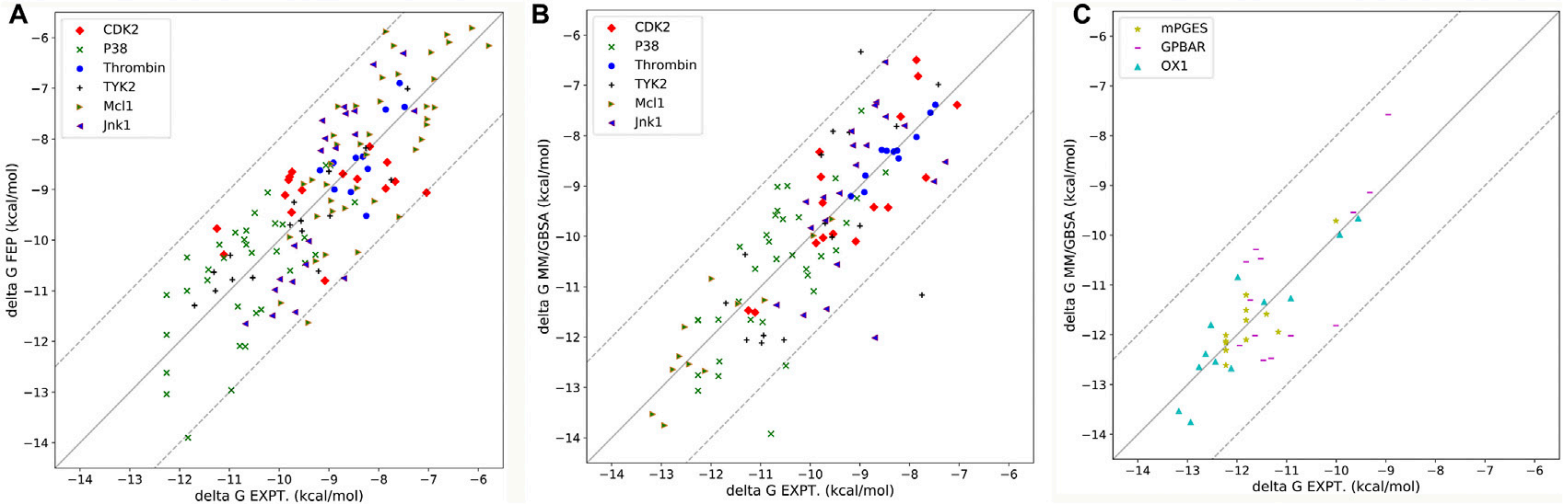
**(A)** Correlation between FEP-predicted binding free energies and experimental data for six soluble protein systems. Data is reported by Wang et al., 2015. **(B)** Correlation between MM/GBSA-predicted binding free energies and experimental data for six soluble protein systems. **(C)** Correlation between MM/GBSA-predicted binding free energies and experimental data for three membrane protein systems.

After optimizing the parameters of MM/PB(GB)SA calculation, the highest R(s) in all nine systems with different parameters were shown in Table 3 and Figure 4. For six soluble protein systems, only three of 140 FEP(OPLS2.1)-predicted binding free energies (Figure 4A) and five of 140 MM/GBSA-predicted binding free energies (Figure 4B) of studied ligands deviated from their experimental free energies by more than 2 kcal/mol. For all three membrane protein systems, no studied ligands deviated from their experimental free energies by more than 2 kcal/mol (Figure 4C).

**TABLE 3:** The R-value of docking, MM/GBSA, MM/PBSA and FEP.

Methods	System								
	CDK2	P38	Thrombin	Mcl1	Jnk1	Tyk2	mPGES	GPBAR	OX1
Docking^a^	−0.56	0.14	0.53	0.59	0.65	0.79	0.72	0.71	0.4
MM/GBSA^b^	**0.82**	0.70	**0.96**	**0.78**	0.55	0.66	0.86	0.7	**0.91**
MM/PBSA^b^	0.58	0.18	0.81	0.70	0.56	0.87	**0.89**	**0.79**	0.82
MM/PBSA/WSAS^c^	0.52	0.22	0.62	0.33	0.04	0.54			
MM/PBSA/ELIE^c^	0.75	0.67	0.63	0.41	0.60	0.62			
FEP(OPLS2.1)^a^	0.48	0.65	0.71	0.77	**0.85**	0.89			
FEP(OPLS3e)^d^	0.57	0.75	0.53	0.52	0.60	0.84			
FEP (XFEP)^e^	0.75	**0.86**	0.22	0.55	0.62	**0.91**			

^a^ Reported by ref (Wang et al., 2015).

^b^ Our results.

^c^ Reported by ref (Hao et al., 2020), ELIE: extended linear interaction energy method.

^d^ Reported by ref (Roos et al., 2019).

^e^ Reported by ref (Lin et al., 2021).

The bold value means the highest R-value with different methods in specific systems.

The Pearson correlation coefficients of docking, MMPBSA and FEP from other publications were also compared in Table 3. In general, the accuracy of MM/GBSA and FEP(s) was similar, both surpassing docking with Glide software in terms of ΔG prediction. Moreover, the performance of MM/GBSA after optimizing the parameters in our study was better that the counterparts of MM/PBSA/WSAS and MM/PBSA/ELIE, which was reported in ref (Hao et al., 2020). MM/GBSA had superiority over the three FEP methods in CDK2, Mcl1 and thrombin systems. The Pearson correlation coefficients with MM/GBSA methods in the CDK2, Mcl1 and thrombin systems were 0.82 0.78 and 0.96, which was higher than that with any other methods in Table 3. The Pearson correlation coefficients with FEP(OPLS2.1) in the Jnk1 system was 0.85, which was also higher than that with other methods. The Pearson correlation coefficients with XFEP were 0.86 and 0.91 in P38 and TYK2 systems respectively. Moreover, the prediction binding energies and experimental binding energies were also recorded in Supplementary Table S6.

## Discussion

The positive performance of MM/PB(GB)SA demonstrates that MM/PB(GB)SA is a powerful tool to predict the binding free energies between protein and ligands, which depends on the parameters, including system properties, ligand charge methods, protein force fields and others. The charge method is an important parameter affecting the performance of MM/PB(GB)SA. From the aspect of average obvious R (the Pearson correlation coefficient in Supplementary Table S1), RESP charges yield the optimal predictive power in both MM/PBSA and MM/GBSA calculations than the semi-empirical method (AM1-BCC) and empirical method (CGenFF). Besides, the different performance of RESP_DFT and RESP_HF suggests that a higher-level basis set contributes to the accuracy of MM/PB(GB)SA. Although the CGenFF charge method is not brilliant in average R, it shows the highest R in p38, thrombin and GPBAR systems with MM/PBSA model. That is because the accuracy of the CGenFF charge method depends on the similarity of the ligand substructure and reference substructure in the CGenFF database. The different performance of the FF99SB force field and CHARMM force field is in line with our expectations because MM/PB(GB)SA models are developed based on AMBER force fields. Interestingly, the difference in predictive power between the FF99SB force field and the CHARMM force field shows little significance in most cases. In p38, thrombin and GPBAR systems, the MM/PBSA even shows the highest obvious R with the combination of the CGenFF charge method and the CHARMM force field. This suggests that no specific force field is suitable for MM/PB(GB)SA calculation in all systems.

The accuracies of MM/PB(GB)SA in soluble protein systems and membrane protein systems were compared here. As the implicit water model and implicit membrane model were used in soluble protein systems and membrane protein systems respectively, the performance of MM/PB(GB)SA with two kind of models was different. Moreover, the performance of MM/PB(GB)SA in the whole system (soluble protein systems and membrane protein systems) was worse than the counterparts in soluble protein systems or in membrane protein systems, indicating that the soluble protein systems and membrane protein systems should be processed separately in MM/PB(GB)SA calculations.

The accuracies of MM/GBSA and MM/PBSA were also compared in this paper. Originally, MM/GBSA is considered to be the approximated form of MM/PBSA to some extent. However, MM/PBSA does not perform significantly better than MM/GBSA in our work as well as other publications (Hou et al., 2011a; Chen et al., 2016). This might be because MM/PBSA model is more sensitive to the parameter set, including the dielectric constant. Hou et al. (2011a) pointed out that the Parse parameter set performs badly in solvation free energy calculation for complex functional groups, such as residue side chain analogs. Therefore, MM/GBSA model is recommended in this paper because of its high accuracy, robustness and low computing resource cost.

The RESP charge method significantly improves the performance of MM/PB(GB)SA in our study, which is quite unexpected but consistent with the previous study (Xu et al., 2013). Previously, it was considered to be a weak factor in the accuracy of MM/PB(GB)SA due to the limitation of computing resources. In fact, the correctness of RESP charge itself sharply depends on the choice of density functions and basis sets (Schauperl et al., 2020). Nowadays, the growing computing resources makes computing RESP charge with a more expensive combination of density functions and basis sets possible, further improving the accuracy of MM/PB(GB)SA calculation. That should also be the reason why RESP_DFT performs better than RESP_HF in some systems.

The simulation of halogen bonds is also evaluated in this study. The role of halogen atoms in halogen bonds is divided into halogen bond acceptor and donor. Different from the halogen bonds with halogen atoms as acceptors, the halogen bonds with halogen atoms as donors cannot be simulated directly. As halogen bond acceptors are electron enriched atoms (such as oxygen and nitrogen), they tend to repel halogen atoms in molecular dynamics simulation. However, an electrophilic region (σ-hole) can be generated outside the halogen atoms in ligands with halogen atoms, which can attract negative charge atoms. To address the problem, extra points with positive charges are added to simulate halogen bonds. That is because the extra points supply positive charges between halogen bond acceptors and halogen atoms in halogen bonds where halogen atoms act as halogen bond donors. And the halogen atoms can be close to halogen bond acceptors by attracting the extra points between them. In our study, ligands can form halogen bonds with the carboxylic acid group of ASP86 in CDK2 protein and the main chain oxygen atom of GLY219 in thrombin protein. As a result, the MM/PB(GB)SA performs better by adding extra points. The study of halogen bonds suggests that extra points benefit the performance of MM/PB(GB)SA in the systems which can form halogen bonds.

The MM/GBSA models supply fast and low-costing methods to predict the ligand binding free energy. The performance of different GB models is compared in our testing cases. As a result, the difference among GBHCT, GBOBC, GBOBC2, and GBneck models is slight. GBneck2 shows the worst performance in all three membrane-bound protein systems, which is out of our expectations. That may be because the GBneck2 model is more sensitive to radii setts. In the Amber reference manual, mbondi3 radii are recommended with GBneck2, which is not adapted in our simulation.

As membrane-bound proteins are embedded in lipid bilayers, nonpolar solvation-free energy is also important in the ligand binding process. Two different methods computing nonpolar solvation energy are implemented in the MMPBSA. py program. In the first method (inp = 1), nonpolar solvation free energy linearly depends on the solvent-accessible surface area. In the second method (inp = 2), nonpolar solvation free energy consists of the cavity term and the dispersion term, which are also related to the solvent accessible surface area. As a result, the first method (inp = 1) performs better in the mPGES system, but worse in GPBAR and OX1 systems. It suggests that no nonpolar solvation free energy computing method is generally better in MM/PBSA calculations. The reason why those methods perform differently in the three systems might be the different positions of the binding pocket. The binding modes of those three systems are shown in Figure 3. In mPGES systems (Figures 3B,E), the ligands bind at the interface of protein and lipids. Therefore, the ligands show strong interaction with both protein and lipids. In GPBAR and OX1 systems (Figures 3C,D,F,G), the ligands bind at the center of protein and are surrounded by amino acid residues. Therefore, the ligands show weak interaction with lipids.

In membrane-bound protein systems, binding pockets are surrounded by lipids and residues. Therefore, the membrane dielectric constant and internal dielectric constant both play important roles in MM/PBSA calculations. In our study, a higher membrane dielectric constant (higher than 3) contributes to the performance of MM/PBSA calculation for most membrane-bound protein systems. Notably, a higher membrane dielectric constant makes MM/PBSA model performs markedly better in mPGES systems. However, the membrane dielectric constant slightly affects the prediction accuracy of MM/PBSA in GPBAR and OX1 systems. The reason for the different performance of the membrane dielectric constant in different systems is the position of binding pocket. The binding pocket of mPGES protein is next to the membrane (Figures 3B,E), while the binding pockets in GPBAR and OX1 systems are only surrounded by protein (Figures 3C,D,F,G). Our findings are consistent with previous study (Greene et al., 2016). In contrast, proteins show stronger interaction with ligands. Thus, the performance of MM/PBSA is affected by the internal dielectric constant. Similar to the membrane dielectric constant, a higher internal dielectric constant also contributes to the performance of MM/PBSA calculation. This result is also supported by the previous study (Sun et al., 2014). In conclusion, the recommended values of membrane dielectric constant (7.0) and internal dielectric constant (20.0) are appropriate in those three membrane-bound protein systems. It might be feasible for other membrane-bound protein systems as well.

Finally, we also compare the performance of MM/PB(GB)SA with docking and FEP. Compared to docking, MM/PB(GB)SA is more accurate but computing resources consuming. Compared to the FEP method, MM/PB(GB)SA shows similar ΔG prediction ability and costs much less computational resources (Wang et al., 2019). Therefore, considering the balance between accuracy and computing resources, docking is suggested for massive drug virtual screening, whereas MM/GBSA is suggested for small-scale virtual screening and lead compound optimization. In addition, the great performance of MM/PB(GB)SA in CDK2 and thrombin systems shows that the addition of extra points in MD simulation results in a higher accuracy of protein-ligand binding energy predictions by modeling halogen bonds.

In summary, we have systemically investigated the effect of the ligand charge method, protein force field, extra point, GB model, nonpolar optimization method, internal dielectric constant and membrane dielectric constant on the performance of MM/PB(GB)SA using 177 ligands and nine proteins. In terms of the ligand charge method, quantum chemistry supplies a more accurate method than the semi-empirical and empirical methods. A higher-level basis set in QM calculation contributes to the accuracy of MM/PB(GB)SA. In addition, adding extra points improves the performance of MM/PB(GB)SA in the systems that can form halogen bonds. No obvious transformation of the accuracy of MM/PB(GB)SA is found with different protein force fields. No GB model shows the best performance in all systems and a modified GBneck2 model (igb = 8) shows the worst performance in all three systems because the GBneck2 model is more sensitive with radii setts. Finally, a higher interior dielectric constant and membrane dielectric constant are necessary to improve the rescoring accuracy of MM/PBSA calculations. The recommended values of membrane dielectric constant (7.0) and internal dielectric constant (20.0) are appropriate in those three membrane-bound protein systems and may be suitable for other membrane-bound protein systems. After optimizing appropriate parameters, the performance of MM/PB(GB)SA with docking and free energy perturbation (FEP) are compared in Table 3. MM/PB(GB)SA shows quite remarkable performance with FEP and better performance with docking in accuracy. All in all, MM/PB(GB)SA shows powerful ranking capability in all nine systems. Meanwhile, the stability and robustness of MM/PB(GB)SA are determined by the parameters mentioned above.

## Data Availability

The original contributions presented in the study are included in the article/Supplementary Material, further inquiries can be directed to the corresponding author. The input structures for MD simulation and a tutorial for adding extra points are included in ourrepository (https://github.com/shiyu-wangbyte/MM-PB-GB-SA_Benchmarks).
