# Supplementary material for: MM/PB(GB)SA benchmarks on soluble proteins and membrane proteins
Source: Front Pharmacol. 2022 Dec 1;13:1018351. doi: 10.3389/fphar.2022.1018351 (PMC9751045; doi:10.3389/fphar.2022.1018351)
Supplement: Supplementary file 1 [file DataSheet1.pdf]

MM/PB(GB)SA Benchmarks on Soluble Proteins and Membrane Proteins
Supporting Information

Authors: Shiyu Wang[1,2,3], Xiaolin Sun[1,3], Wenqiang Cui[1,3], Shuguang Yuan[1,3,4*]

1Research Center for Computer-Aided Drug Discovery, Shenzhen Institute of Advanced Technology, Chinese Academy of Sciences, Shenzhen, China,
2College of Chemical Science, University of Chinese Academy of Sciences, Beijing, China
3 AlphaMol-SIAT joint laboratory, Shenzhen, China
4 Faculty of Chemistry, University of Warsaw, Warsaw, Poland

Details about R and MAE calculation

The correlation coefficient (R) is defined as the ratio of covariance to respective variance, varying from -1 to 1. For vector ΔG_{exp}=[$x_1, x_2, \ldots\ldots, x_n$], $\Delta G_{compute}$=[$y_1, y_2, \ldots\ldots, y_n$], $\Delta G_{pred} = [z_1, z_2, \ldots\ldots, z_n]$, R can be computed with the following formula:

$$R = \frac{\sum_{i=1}^{n}(x_i - \bar{x})(y_i - \bar{y})}{\sqrt{\sum_{i=1}^{n}(x_i - \bar{x})^2}\sqrt{\sum_{i=1}^{n}(y_i - \bar{y})^2}}$$

where, $\bar{x}$, $\bar{y}$ are mean values of elements in X and Y.

We define $\Delta G_{compute} = k\Delta G_{exp} + b$. Therefore, k and b can be computed with the following formulas:

$$k = \frac{\sum_{i=1}^{n} x_i y_i - n\,\bar{x}\,\bar{y}}{\sum_{i=1}^{n} x_i^2 - n\,\bar{x}^2}$$

$$b = \bar{y} - k\,\bar{x}$$

In our study, the values of k and b were calculated by numpy which is a python module for scientific computation. Therefore, the predicted ΔG can be liner fitted by the following formula:

$$\Delta G_{pred} = \frac{\Delta G_{compute} - b}{k}$$

And, the Mean Absolute Error (MAE) is defined as the error between $\Delta G_{pred}$and ΔG_{exp}:

$$\text{MAE}=\frac{\sum_{i=1}^{n}|z_i - x_i|}{n}$$

The reason why we liner fit the predicted ΔG before MAE calculation is that the relative value of ΔG is more meaningful that its absolute value. The ΔG_{exp} was estimated via $RTlnK$ (equation 2) in our study, where, k varies with different experimental methods. Thus, ΔG_{exp} is not an absolute value. Moreover, MM/PB(GB)SA is used to rank molecules in real drug design process, indicating that the relative value is more meaningful.

Supplementary Table 1. The Pearson correlation coefficient (R) and mean absolute error (MAE) between MM/PB(GB)SA-predicted binding free energies and experimental data based on different ligand charge methods and protein force fields.

		**obs. R (higher is better)**						**MAE (lower is better)**					
		RESP_DFT		RESP_HF		AM1_BCC	CGenFF	RESP_DFT		RESP_HF		AM1_BCC	CGenFF
		FF99SB	CHARMM	FF99SB	CHARMM	FF99SB	CHARMM	FF99SB	CHARMM	FF99SB	CHARMM	FF99SB	CHARMM
**Soluble Protein Systems**	MM/GBSA	0.06	0.11	0.01	0.06	-0.04	-0.31	118.35	53.81	609.93	105.53	26.11	3.62
	MM/PBSA	-0.36	-0.41	-0.44	-0.41	-0.57	-0.57	23.07	21.59	17.88	22.18	1.66	1.62
**Membrane Protein Systems**	MM/GBSA	0.47	0.50	0.45	0.49	**0.57**	0.38	8.59	7.70	9.70	7.58	1.29	1.93
	MM/PBSA	**0.45**	0.44	0.45	0.42	0.44	0.30	7.41	7.17	7.07	8.03	1.82	2.55
**All systems**	MM/GBSA	0.33	**0.34**	0.33	0.32	**0.40**	-0.17	21.01	17.51	19.21	19.38	3.22	7.84
	MM/PBSA	**0.17**	0.08	0.13	0.09	0.10	-0.09	62.23	133.41	76.06	124.82	13.77	14.7

Supplementary Table 2. The Pearson correlation coefficient (R) and mean absolute error (MAE) between MM/PB(GB)SA-predicted binding free energies and experimental data based on different GB models.

		obs. R (higher is better)							MAE (lower is better)						
		RESP_DFT		RESP_HF		AM1_BCC	CGenFF	Average	RESP_DFT		RESP_HF		AM1_BCC	CGenFF	Average
		FF99SB	CHARMM	FF99SB	CHARMM	FF99SB	CHARMM		FF99SB	CHARMM	FF99SB	CHARMM	FF99SB	CHARMM	
mPGES	igb=1	0.84	0.86	0.82	0.81	0.82	0.70	0.81	0.31	0.29	0.34	0.35	0.32	0.59	0.36
	igb=2	0.84	0.85	0.83	0.82	0.84	0.58	0.79	0.32	0.29	0.32	0.33	0.30	0.77	0.38
	igb=5	0.83	0.84	0.85	0.82	0.85	0.08	0.73	0.33	0.29	0.30	0.34	0.29	6.85	1.25
	igb=7	0.80	0.84	0.84	0.80	0.84	0.69	0.80	0.36	0.34	0.30	0.38	0.31	0.55	0.37
	igb=8	0.68	0.70	0.72	0.68	0.80	-0.19	**0.57**	0.49	0.46	0.43	0.53	0.36	2.87	0.84
GPBAR	igb=1	0.68	0.66	0.69	0.69	0.68	0.66	0.68	0.79	0.92	0.78	0.88	0.76	0.90	0.83
	igb=2	0.69	0.68	0.70	0.69	0.70	0.64	0.68	0.84	0.86	0.81	0.91	0.78	0.90	0.83
	igb=5	0.66	0.68	0.67	0.69	0.67	0.62	0.66	0.95	0.85	0.90	0.89	0.90	0.96	0.89
	igb=7	0.70	0.67	0.68	0.64	0.70	0.65	0.70	0.89	0.95	0.95	1.09	0.89	0.86	0.87
	igb=8	0.43	0.12	0.34	0.18	0.29	0.23	**0.27**	1.70	7.07	2.14	3.95	2.52	3.65	3.41
OX1	igb=1	0.82	0.58	0.60	0.60	0.48	0.75	0.62	0.64	1.38	1.15	1.20	1.62	0.91	1.22
	igb=2	0.85	0.64	0.66	0.69	0.63	0.77	0.70	0.53	1.20	1.04	0.94	1.12	0.84	0.96
	igb=5	0.85	0.64	0.58	0.70	0.69	0.75	0.71	0.49	1.18	1.30	0.92	0.97	0.85	0.94
	igb=7	0.91	0.80	0.87	0.83	0.78	0.79	**0.81**	0.39	0.69	0.57	0.64	0.80	0.78	**0.68**
	igb=8	0.88	0.78	0.83	0.80	0.50	0.81	**0.73**	0.55	0.72	0.65	0.71	1.54	0.70	0.94

Supplementary Table 3. The Pearson correlation coefficient (R) and mean absolute error (MAE) between MM/PB(GB)SA-predicted binding free energies and experimental data based on different nonpolar optimization methods.

		**obs. R (higher is better)**							**MAE (lower is better)**						
		RESP_DFT		RESP_HF		AM1_BCC	CGenFF	Average	RESP_DFT		RESP_HF		AM1_BCC	CGenFF	Average
		FF99SB	CHARMM	FF99SB	CHARMM	FF99SB	CHARMM		FF99SB	CHARMM	FF99SB	CHARMM	FF99SB	CHARMM	
mPGES	inp=1	0.87	0.87	0.87	0.85	0.89	0.47	**0.81**	0.28	0.31	0.26	0.31	0.26	0.90	0.37
	inp=2	0.80	0.80	0.77	0.67	0.87	-0.15	0.64	0.35	0.41	0.35	0.59	0.27	3.58	0.86
GPBAR	inp=1	0.69	0.70	0.67	0.70	0.62	0.71	**0.67**	0.86	0.93	0.91	0.88	1.00	0.75	0.91
	inp=2	0.69	0.71	0.67	0.69	0.63	0.76	**0.66**	0.86	0.89	0.94	0.91	0.95	0.62	0.96
OX1	inp=1	0.66	0.57	0.61	0.63	0.49	0.64	0.59	1.03	1.29	1.12	1.22	1.57	1.09	1.24
	inp=2	0.73	0.71	0.70	0.66	0.57	0.66	**0.66**	0.75	0.78	0.95	1.14	1.36	1.19	1.06

Supplementary Table 4. The Pearson correlation coefficient (R) and mean absolute error (MAE) between MM/PB(GB)SA-predicted binding free energies and experimental data based on different membrane dielectric constants.

		**obs. R (higher is better)**							**MAE (lower is better)**						
		RESP_DFT		RESP_HF		AM1_BCC	CGenFF	Average	RESP_DFT		RESP_HF		AM1_BCC	CGenFF	Average
		FF99SB	CHARMM	FF99SB	CHARMM	FF99SB	CHARMM		FF99SB	CHARMM	FF99SB	CHARMM	FF99SB	CHARMM	
mPGES (inp=1)	emem=1	0.81	0.86	0.85	0.83	0.88	0.47	**0.80**	0.36	0.32	0.27	0.34	0.27	0.90	**0.39**
	emem=3	0.85	0.86	0.86	0.84	0.89	0.47	0.81	0.30	0.31	0.25	0.32	0.26	0.89	0.37
	emem=5	0.86	0.87	0.87	0.85	0.89	0.47	0.81	0.28	0.31	0.25	0.31	0.26	0.90	0.37
	emem=7	0.87	0.87	0.87	0.85	0.89	0.47	0.81	0.28	0.31	0.25	0.31	0.26	0.90	0.37
	emem=9	0.87	0.87	0.87	0.85	0.89	0.47	0.81	0.27	0.31	0.26	0.30	0.26	0.91	0.36
GPBAR (inp=2)	emem=1	0.69	0.71	0.67	0.68	0.63	0.76	0.66	0.86	0.89	0.94	0.94	0.94	0.62	0.97
	emem=3	0.69	0.71	0.67	0.69	0.63	0.76	0.66	0.86	0.89	0.94	0.92	0.94	0.62	0.96
	emem=5	0.69	0.71	0.67	0.69	0.63	0.76	0.66	0.86	0.89	0.94	0.92	0.93	0.62	0.96
	emem=7	0.69	0.71	0.67	0.69	0.63	0.76	0.66	0.86	0.89	0.94	0.91	0.95	0.62	0.96
	emem=9	0.69	0.71	0.67	0.69	0.63	0.76	0.66	0.86	0.89	0.94	0.91	0.93	0.62	0.96
OX1 (inp=2)	emem=1	0.73	0.71	0.70	0.66	0.57	0.66	0.66	0.75	0.78	0.95	1.13	1.35	1.19	1.06
	emem=3	0.73	0.71	0.70	0.66	0.57	0.66	0.66	0.75	0.78	0.95	1.14	1.36	1.19	1.06
	emem=5	0.73	0.71	0.70	0.66	0.57	0.66	0.66	0.75	0.78	0.95	1.14	1.36	1.19	1.06
	emem=7	0.73	0.71	0.70	0.66	0.57	0.66	0.66	0.75	0.78	0.95	1.14	1.36	1.19	1.06
	emem=9	0.72	0.71	0.70	0.66	0.57	0.66	0.66	0.75	0.78	0.95	1.14	1.36	1.20	1.06

Supplementary Table 5. The Pearson correlation coefficient (R) and mean absolute error (MAE) between MM/PB(GB)SA-predicted binding free energies and experimental data based on different internal dielectric constants.

		obs. R (higher is better)							MAE (lower is better)						
		RESP_DFT		RESP_HF		AM1_BCC	CGenFF	AVERAGE	RESP_DFT		RESP_HF		AM1_BCC	CGenFF	AVERAGE
		FF99SB	CHARMM	FF99SB	CHARMM	FF99SB	CHARMM		FF99SB	CHARMM	FF99SB	CHARMM	FF99SB	CHARMM	
mPGES (inp=1)	indi=1	0.71	0.76	0.75	0.68	0.75	-0.17	**0.60**	0.50	0.46	0.42	0.51	0.41	3.10	0.85
	indi=5	0.85	0.86	0.85	0.81	0.88	0.37	0.78	0.30	0.31	0.27	0.35	0.27	1.27	0.44
	indi=10	0.86	0.87	0.86	0.83	0.88	0.44	0.80	0.29	0.31	0.26	0.33	0.26	1.01	0.39
	indi=20	0.87	0.87	0.87	0.85	0.89	0.47	**0.81**	0.28	0.31	0.25	0.31	0.26	0.90	0.37
	indi=30	0.87	0.87	0.87	0.86	0.89	0.48	0.82	0.27	0.31	0.25	0.30	0.26	0.87	0.36
GPBAR (inp=2)	indi=1	0.37	0.59	0.85	0.06	0.41	0.61	**0.46**	2.15	1.21	0.48	13.97	1.57	1.07	3.23
	indi=5	0.73	0.72	0.72	0.68	0.65	0.79	0.69	0.76	0.82	0.78	0.95	0.87	0.60	0.87
	indi=10	0.71	0.72	0.69	0.69	0.64	0.77	0.67	0.82	0.86	0.88	0.91	0.91	0.60	0.92
	indi=20	0.69	0.67	0.67	0.69	0.63	0.76	0.66	0.86	0.96	0.94	0.91	0.95	0.62	0.97
	indi=30	0.68	0.71	0.66	0.69	0.63	0.75	0.66	0.88	0.90	0.96	0.92	0.95	0.63	0.98
OX1 (inp=2)	indi=1	0.82	0.30	0.47	0.56	0.46	0.67	**0.49**	0.69	2.69	1.68	1.41	1.89	1.07	2.12
	indi=5	0.79	0.60	0.74	0.67	0.59	0.69	0.66	0.63	1.09	0.86	1.16	1.33	1.15	1.09
	indi=10	0.75	0.65	0.72	0.67	0.58	0.67	0.66	0.70	0.89	0.90	1.14	1.34	1.17	1.06
	indi=20	0.73	0.71	0.70	0.66	0.57	0.66	0.66	0.75	0.78	0.95	1.14	1.36	1.19	1.06
	indi=30	0.72	0.68	0.69	0.66	0.56	0.65	0.65	0.77	0.81	0.97	1.14	1.37	1.20	1.07

Supplementary Table 6. The experimental binding energy and prediction binding energy of complexes.

Target	PDB	ligand	ΔG MM/GBSA (kcal/mol)	ΔG EXPT (kcal/mol)
CDK2	1H1Q	17	-7.04	-7.39
		20	-8.72	-9.42
		21	-7.83	-6.82
		22	-7.86	-6.5
		26	-8.43	-9.43
		28	-11.11	-11.51
		29	-9.88	-10.13
		30	-9.81	-8.32
		31	-9.54	-9.95
		32	-9.75	-9.33
		1h1q	-8.18	-7.62
		1h1r	-7.67	-8.83
		1h1s	-11.25	-11.47
		1oi9	-9.74	-10.03
		1oiu	-9.08	-10.1
		1oiy	-9.79	-8.82
P38	3FLY	p38a_3flz	-9.49	-8.85
		p38a_2n	-11.85	-12.78
		p38a_2c	-10.46	-9
		p38a_3flq	-9.93	-11.09
		p38a_2aa	-9.27	-9.74
		p38a_2o	-10.49	-12.57
		p38a_2v	-8.97	-7.51
		p38a_2t	-11.85	-11.65
		p38a_3fly	-11.42	-10.21
		p38a_3fmk	-11.83	-12.49
		p38a_2g	-10.66	-9.02
		p38a_2p	-11.2	-11.65
		p38a_2i	-10.05	-10.77
		p38a_2l	-12.26	-12.76
		p38a_2j	-10.08	-10.65
		p38a_2ff	-11.44	-11.29
		p38a_2s	-11.11	-10.65
		p38a_2z	-10.36	-10.37
		p38a_2ee	-12.26	-13.06
		p38a_2u	-12.26	-11.66
		p38a_3fln	-10.88	-9.97
		p38a_2e	-10.7	-9.58
		p38a_2f	-8.48	-8.73
		p38a_3flw	-10.68	-10.45

		p38a_2k	-10.55	-9.66
		p38a_2x	-10.23	-9.62
		p38a_2q	-12.26	-11.67
		p38a_2gg	-10.66	-9.49
		p38a_2y	-9.78	-10.4
		p38a_2r	-10.79	-13.92
		p38a_2bb	-9.06	-9.24
		p38a_2m	-10.96	-11.7
		p38a_3fmh	-10.83	-10.11
		p38a_2h	-9.48	-10.28
Thrombin	2ZFF	1d	-8.25	-9.2
		5	-7.58	-9.12
		3b	-7.86	-8.79
		1b	-8.46	-8.28
		7a	-8.22	-8.3
		6a	-9.18	-8.32
		6e	-8.91	-8.29
		3a	-8.32	-8.45
		1a	-7.48	-8.03
		1c	-8.56	-7.54
		6b	-8.89	-7.39
Tyk2	4GIH	ejm_31	-9.54	-7.91
		ejm_42	-9.78	-8.38
		ejm_43	-8.26	-7.81
		ejm_44	-7.42	-6.98
		ejm_45	-9.56	-10.01
		ejm_46	-11.31	-10.36
		ejm_47	-9.7	-9.74
		ejm_48	-9	-9.79
		ejm_49	-7.75	-11.16
		ejm_50	-8.98	-6.33
		ejm_54	-10.53	-12.06
		ejm_55	-9.21	-7.93
		jmc_23	-11.7	-11.32
		jmc_27	-11.28	-12.06
		jmc_28	-10.98	-12.12
		jmc_30	-10.94	-11.97
mPGES	5TL9	6	-11.82	-11.71
		7	-11.41	-11.58
		8	-11.82	-11.2
		9	-12.22	-12.01
		10	-12.22	-12.61
		11	-12.22	-12.13

		12	-11.82	-11.5
		13	-11.17	-11.94
		14	-11.82	-12.1
		15	-12.22	-12.31
		16	-12.22	-12.16
		5TL9	-10.01	-9.71
GPBAR	7CFM	20	-10.01	-11.81
		23	-11.62	-10.28
		19	-11.64	-12.02
		21	-11.82	-10.53
		18	-11.31	-12.48
		5	-11.47	-12.51
		22	-11.52	-10.47
		17	-11.95	-12.22
		16	-11.73	-11.3
		14	-10.92	-12.02
		13	-9.65	-9.54
		15	-9.32	-9.14
		12	-8.95	-7.58
OX1	4ZJ8	29	-9.93	-9.99
		30	-11.45	-11.34
		31	-9.56	-9.66
		32	-12.53	-11.8
		33	-12.63	-12.38
		34	-13.17	-13.53
		37	-12.44	-12.54
		38	-10.92	-11.27
		39	-12.77	-12.65
		40	-11.99	-10.84
		41	-12.93	-13.75
		42	-12.12	-12.68
		23472	-8.35	-8.646
		23474	-7.95	-9.112
		23480	-10.62	-8.718
		23470	-8.13	-7.723
		23486	-13	-12.47
		20669(2qbr)	-8.07	-8.607
		23482	-9.8	-10.007
		23483	-10.89	-9.875
		23473	-8.13	-8.389
		20670(2qbs)	-8.69	-9.083
		23468	-7.94	-7.752
		23475	-7.68	-8.718

JNK1	2GMX	18629-1	-8.39	-8.67377
		18634-1	-11.74	-9.99323
		18628-1	-7.2	-8.70259
		18660-1	-8.09	-8.70259
		18624-1	-8.47	-8.49337
		18633-1	-8.82	-9.1743
		18635-1	-10.63	-7.28607
		17124-1	-8.22	-9.67592
		18625-1	-7.38	-8.105
		18659-1	-11.05	-9.46522
		18637-1	-11.32	-10.1417
		18638-1	-8.86	-10.0931
		18652-1	-9.74	-10.683
		18627-1	-7.77	-8.4831
		18658-1	-10.19	-9.6994
		18630-1	-9.46	-9.14236
		18639-1	-10.77	-9.74073
		18631-1	-9.63	-9.41382
		18632-1	-9.97	-9.08324
		18636-1	-5.71	-7.51232
		18626-1	-7.72	-8.87254
MCL1	4HW3	35	-8.02	-8.81265
		41	-6.75	-7.12925
		31	-6.96	-7.91952
		49	-7.06	-8.36146
		52	-8.56	-9.22585
		32	-5.8	-6.58302
		57	-8.75	-9.03887
		43	-7.72	-7.0347
		65	-10.18	-8.40734
		26	-8.58	-8.23584
		48	-6.97	-6.66262
		44	-8.92	-8.67338
		29	-6.78	-6.93941
		53	-9.87	-9.96487
		54	-8.93	-9.77998
		37	-7.36	-8.95356
		56	-9.29	-9.25808
		30	-6.87	-7.85321
		64	-9.63	-9.49979
		38	-8.58	-7.01901
		40	-6.87	-7.25303
		33	-6.33	-6.87524

		36	-8.06	-8.17902
		62	-8.86	-7.95566
		39	-5.35	-7.0268
		63	-10.23	-9.06225
		68	-8.56	-7.68961
		66	-9.37	-8.43167
		58	-10.19	-9.4079
		61	-9.37	-8.07944
		67	-8.81	-7.58092
		27	-4.74	-6.11639
		50	-8.97	-9.3283
		28	-7.36	-6.62149
		47	-6.46	-5.77553
		42	-8.5	-8.89675
		45	-9.52	-8.95356
		23	-8.9	-8.82854
		51	-8.26	-8.44846
		60	-9.07	-8.91509
		34	-6.3	-6.86919
		46	-7.6	-7.60113

Input file for MM/GB(PB)SA with CDK2 complex

```
&general
sys_name="Protein-ligand-complex",   ; system name
startframe=300,                      ; Start extracting snapshots from 300th frame
endframe=500,                        ; Stop extracting snapshots from 500th frame
verbose=2,                           ; Print the number of iterations required to
converge
interval=4,                          ; The offset between two snapshots
/

&gb
igb=5,                               ; GBOBC2 method to use
saltcon=0.150,                       ; 0.15M NaCl
/

&pb
istrng=0.15,                         ; 0.15M NaCl
fillratio=4.0,                       ; The ratio between the longest dimension of the
rectangular finite-difference grid and that of the solute
radiopt=0,                           ; Atomic radii taken from topology files
/
```

Input file for MM/GB(PB)SA with OX1 complex

&general
sys_name="Protein-ligand-Membrane-complex", ; system name
startframe=300, ; Start extracting snapshots from 300th frame
endframe=500, ; Stop extracting snapshots from 500th frame
debug_printlevel=2, ; The level of printing errors
use_sander=1, ; Use sander for energy calculations
interval=2, ; The offset between two snapshots
/

&gb
igb=5, ; GB^{OBC2} method to use
saltcon=0.150, ; 0.15M NaCl
/

&pb
radiopt=0, ; Atomic radii taken from topology files
indi=20.0, ; Internal dielectric constant
istrng=0.150, ; 0.15M NaCl
fillratio=1.25, ; The ratio between the longest dimension of the rectangular finite-difference grid and that of the solute
ipb=1, ; Classical geometric method was used to set up dielectric model
nfocus=1, ; No electrostatic focusing calculation
bcopt=10, ; Periodic boundary condition
eneopt=1, ; Compute total electrostatic energy and forces with P3M procedure
cutfd=7.0, ; Cutoff distance
cutnb=99.0, ; Cutoff distance for Van der Waals interaction
npbverb=1, ; verbose mode in *pbsa* program
solvopt=2, ; Geometric multigrid iterative solvers
inp=2, ; Compute non-polar solvation free energy method[1]
memopt=1, ; Signal for membrane protein
emem=7.0, ; Membrane dielectric constant
mctrdz=60, ; Absolute membrane center in the z-direction
mthick=40, ; Membrane thickness
poretype=1, ; Automatic membrane finding method
maxarcdot=15000 ; Number of arc dots required for solvent accessible surface
/

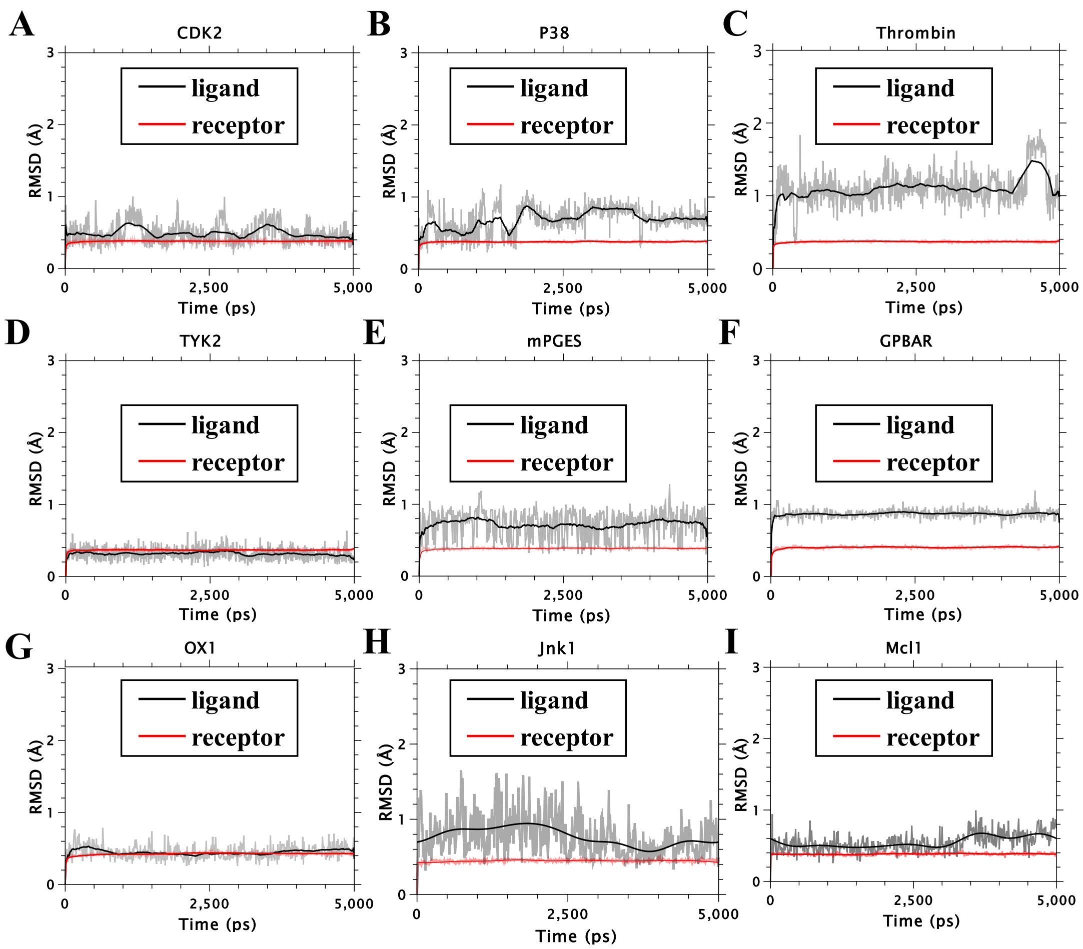

Supplementary Figure 1. RMSD figures for all nine systems studied.

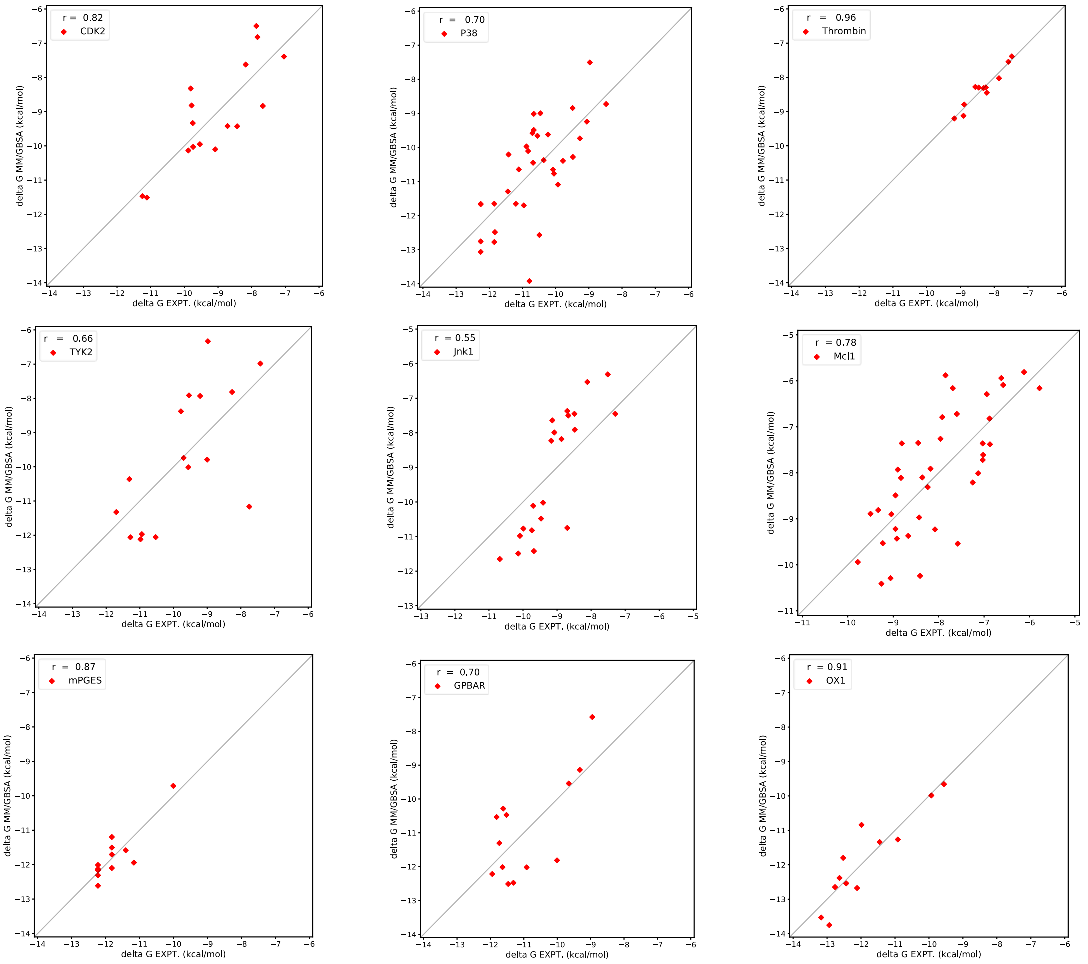

Supplementary Figure 2. Correlation between MM/GBSA-predicted binding free energies and experimental data for all nine systems studied.

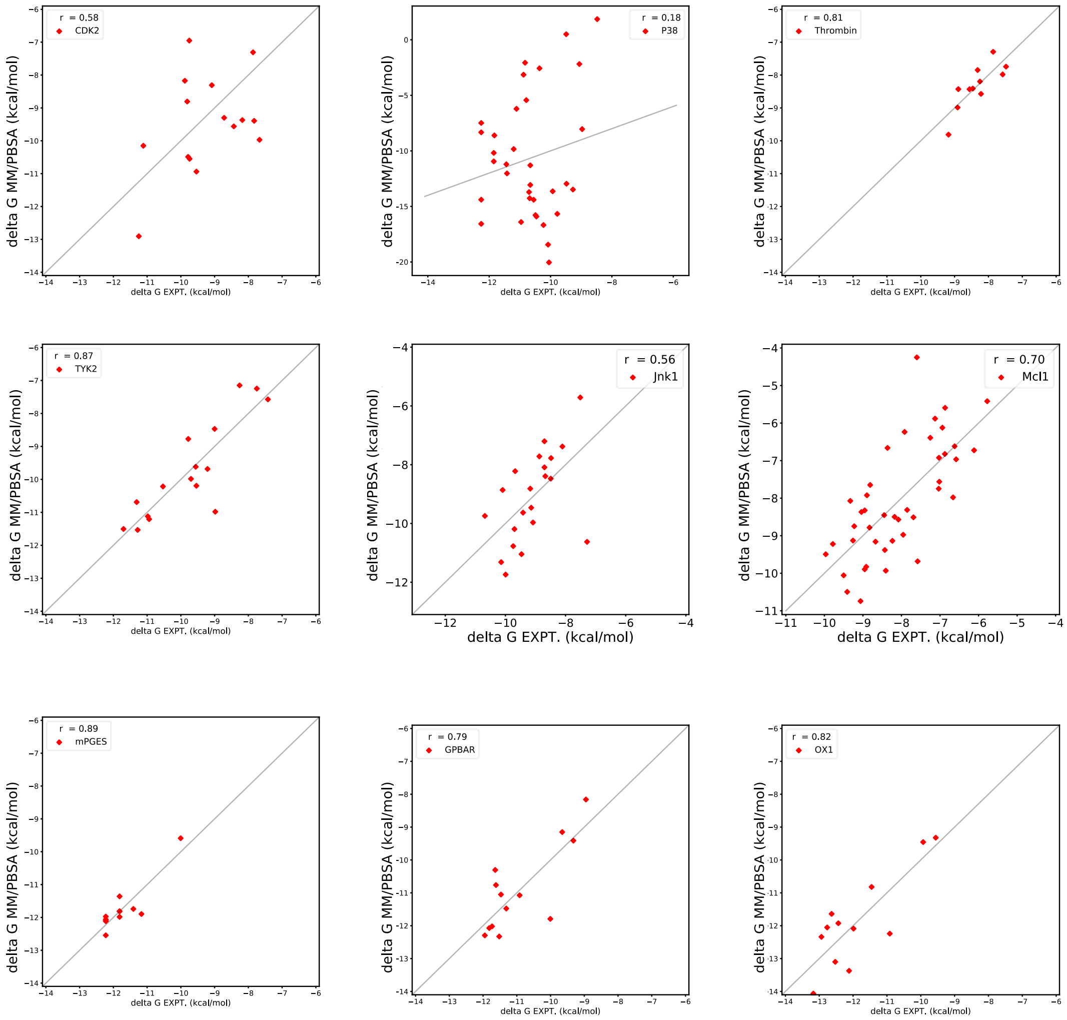

Supplementary Figure 3. Correlation between MM/PBSA-predicted binding free energies and experimental data for all nine systems studied.
